# Approaches to Capture Variance Differences in Rest fMRI Networks in the Spatial Geometric Features: Application to Schizophrenia

**DOI:** 10.3389/fnins.2016.00085

**Published:** 2016-03-07

**Authors:** Shruti Gopal, Robyn L. Miller, Stefi A. Baum, Vince D. Calhoun

**Affiliations:** ^1^Chester F. Carlson Center for Imaging Science, Rochester Institute of TechnologyRochester, NY, USA; ^2^The Mind Research NetworkAlbuquerque, NM, USA; ^3^Faculty of Science, University of ManitobaWinnipeg, MB, Canada; ^4^Department of Electrical and Computer Engineering, University of New MexicoAlbuquerque, NM, USA

**Keywords:** IVA, schizophrenia, spatial variability, resting fMRI

## Abstract

Identification of functionally connected regions while at rest has been at the forefront of research focusing on understanding interactions between different brain regions. Studies have utilized a variety of approaches including seed based as well as data-driven approaches to identifying such networks. Most such techniques involve differentiating groups based on group mean measures. There has been little work focused on differences in spatial characteristics of resting fMRI data. We present a method to identify between group differences in the variability in the cluster characteristics of network regions within components estimated via independent vector analysis (IVA). IVA is a blind source separation approach shown to perform well in capturing individual subject variability within a group model. We evaluate performance of the approach using simulations and then apply to a relatively large schizophrenia data set (82 schizophrenia patients and 89 healthy controls). We postulate, that group differences in the intra-network distributional characteristics of resting state network voxel intensities might indirectly capture important distinctions between the brain function of healthy and clinical populations. Results demonstrate that specific areas of the brain, superior, and middle temporal gyrus that are involved in language and recognition of emotions, show greater component level variance in amplitude weights for schizophrenia patients than healthy controls. Statistically significant correlation between component level spatial variance and component volume was observed in 19 of the 27 non-artifactual components implying an evident relationship between the two parameters. Additionally, the greater spread in the distance of the cluster peak of a component from the centroid in schizophrenia patients compared to healthy controls was observed for seven components. These results indicate that there is hidden potential in exploring variance and possibly higher-order measures in resting state networks to better understand diseases such as schizophrenia. It furthers comprehension of how spatial characteristics can highlight previously unexplored differences between populations such as schizophrenia patients and healthy controls.

## Introduction

The human brain in a complex network of regions that are interconnected structurally and functionally. Interaction between the different regions of the brain and their functioning is known to impact cognition (Casey et al., 2000; Phan et al., 2002; Amodio and Frith, 2006). This has been the primary reason for the focus on examination of the behavior of functionally connected regions of the brain. Such studies have in turn lead to a better understanding of the relationship between functional activation of the brain and an individual's cognitive abilities and the expression of neuropsychiatric symptoms (Alivisatos and Petrides, 1997; Hamilton et al., 2009). However, years of cyto-architechtonic, genetic, and environmental studies show that interaction between different brain regions is highly driven by inter-individual differences at the structural, cellular as well as functional levels. These differences are known to result in cognitive differences manifesting as varied performance in cognitive activities and possibly as varied symptom expression in populations with neuropsychiatric disorders (Zilles and Amunts, 2010, 2013). Furthermore, such variability may go beyond a systemic difference from the mean of the patient population by manifesting as greater variability among the patients. This may possibly present as a spread in anatomical or functional variability in patients relating to the spread in cognitive abilities or symptom expression (Fornito et al., 2008). Such inconsistency in the population characterized by disorders like schizophrenia makes looking at variability potentially meaningful.

Resting state fMRI (rs-fMRI) is used to evaluate regional interactions and allows us to explore functional organization of the brain in the absence of an explicit task or stimuli. Analyses of functional interactions and the differences between populations representing the intrinsic connectivity of the brain employ both model-based as well as data-driven methods (Gold et al., 1998; Calhoun, 2002; Zalesky et al., 2011). The dependence of the observed rs-fMRI signal on non-neural or physiological factors such as shape, size, folding patterns, and location of areas with particular cell types are however a confounding factor since it introduces variability in activation within the population. Traditional methods focus on aligning individual brains to a common space in order to determine similarity or dissimilarity of activation patterns across subjects. Such techniques allow us to compare groups and explore the cognitive architecture of different groups in comparison to healthy individuals.

Inter-subject variability in fMRI data has been shown to be meaningful in previous studies (Frost and Goebel, 2012; Mueller et al., 2013; Zilles and Amunts, 2013; Gopal et al., 2016) through functional variability analyses as well as cytoarchitectonic studies. Studies of variability in the location of functional loci (Sabuncu et al., 2010) and neuroanatomical variability in human brains (Li et al., 2013; Mueller et al., 2013) have provided traction to the concept of including inter-subject variability analyses while looking to differentiate groups. Many studies have also shown that functional variability could be introduced due to environmental influences such as learning or disease (Garavan et al., 2000). A clear conclusion can thus be drawn that incorporating this variability across subjects in a study can provide us with additional information about how environment and experience can affect the brain.

Another important consideration here is that there exists an underlying relationship between the variability across subjects and the spread of activation within a subject. Studies have linked the presence of subject level differences in functional activation patterns to the observance of voxel-level variability in the functional activation patterns of a given subject (Davis et al., 2014). Davis et al. (2014), show that univariate voxel-wise methods are sensitive to variability in the parameters relating within voxel activation to experimental variables between subjects. These observations might stem from the variability in the cortical folding patterns across people or even the variability in the functional subdivisions on the cortical surface. Even if the functional sources were spatially normalized across the subjects, the inter-subject variability of cortical folding perhaps results in inclusion of unique characteristics of the functional activation sources for every subject to result in the acquired fMRI data. These studies strengthen the motivation to study the relationship between inter-subject variability and the variability within the functional subdivisions of the brain. Further exploration of such a relationship might shed new light on how individual differences place a subject on a spectrum of the cognitive performance abilities as well as whether the inter-subject variability is driven by the extent of the sources or functional localization issues.

We can quantify variance in the context of whole-brain multivariate analysis techniques at the level of the estimated source components. Multivariate analysis techniques such as independent vector analysis (IVA) have been established as suitable for data-driven analyses of rs-fMRI data while capturing individual features of each subjects' statistically independent component maps (Fornito et al., 2008; Zilles and Amunts, 2010). Recent studies (Anderson et al., 2012; Ma et al., 2013) provide abundant evidence that IVA captures individual subject variability in spatial patterns (Michael et al., 2014; Rashid et al., 2014), show that functional connectivity networks can be estimated similar to those from GICA, and others substantiate this observation in simulations and in evaluating dynamic functional network connectivity patterns (Adali et al., 2014, 2015; Ma et al., 2014; Laney et al., 2015). Results from our previous study (Gopal et al., 2016) emphasized the presence of subject-level variability in schizophrenia which can efficiently be used as a tool to differentiate patients from healthy controls. Schizophrenia is a complex disorder afflicting a diverse population of patients presenting with a range of symptoms (Ngan and Liddle, 2000; Perlstein et al., 2001; Ngan et al., 2002). The etiology of the disorder is not well understood but appears to involve many different structural as well as functional activation based variations not consistent across the population. The structural inconsistencies might in turn render the functional and cognitive abilities of patients inconsistent stemming from the relationship between structural differences and cognitive abilities of patients (Yao et al., 2015). Schizophrenia is thus a disorder that is particularly well-suited for studies involving analysis of variability of features drawn from brain imaging data. While many studies have focused on analyzing spatial inter-subject variability in different populations (Gao et al., 2014; Gopal et al., 2016; Laney et al., 2015), no known study has utilized whole-brain analysis to study component level variance and explore the geometric source of variability across patients in IVA estimated sources. There have been no studies that utilize higher-order distributional statistics as parameters of component spatial maps so as to explore features such as variance in terms of the size and location of component sources.

This study is thus aimed at exploring whether component-level variability in the extent and voxel amplitude distribution of a component relates to subject-level variability and if this brings to light any new evidence that helps in improving our understanding of schizophrenia. We use simulations to explore if translational variation in functional sources could introduce sufficient population-level variability to be quantifiable using IVA. Furthermore, we introduce measures of spatial component level variability which through simulations allows us to identify one possible origin of variance in IVA components in resting fMRI data that differentiate schizophrenia patients and healthy controls. We hypothesize based on previous studies that schizophrenia patients will have greater variability in the geometry of the estimated sources and expect that this study will provide us renewed direction in terms of differentiating schizophrenia patients from healthy controls.

## Methods

### IVA

IVA is a data-driven algorithm that is used to investigate functional connectivity patterns in the whole brain by identifying statistically independent sources with cross-subject dependencies while retaining individual features of the subjects for further analyses. Studies provide abundant evidence that IVA captures individual subject variability in spatial patterns and others substantiate this observation in simulations and in evaluating dynamic functional network connectivity patterns as well as in large datasets to differentiate schizophrenia patients from healthy controls (Ma et al., 2013, 2014; Michael et al., 2013, 2014; Gopal et al., 2016; Laney et al., 2015). The algorithm models the measured BOLD fMRI signal as a linear combination of the independent activation sources that comprise the measured signal. IVA starts with the same assumption as in GICA that the individual sources of each subject's data are spatially independent but additionally considers statistical dependence of the corresponding sources across other subjects. The demixing of these sources are estimated by minimizing mutual information among source component vectors across subjects. These estimations can be characterized by the following equations:

(1) $$X_{i}=A_{i}\;\times S_{i}$$

(2) $$U_{i}=W_{i}\times X_{i}$$

where *X*_*i*_ is the observed BOLD signal, *A*_*i*_ is the mixing matrix, and *S*_*i*_ are the individual sources that comprise *X*_*i*_. The *W*_*i*_ is the unmixing matrix that represents the inverse of the *A*_*i*_ which is that decomposes the BOLD signal into the component sources *U*_*i*_. The sources *U*_*i*_ are the component sources that are estimated in a manner such that these are matched across the subjects despite the independence. IVA-GL is an adaptation of the IVA algorithm that allows estimation of independent sources using a Gaussian as well as Laplacian density models (Anderson et al., 2012). This model incorporates second as well as higher order dependence among multiple data sets (subjects) into account and thus assumes super-Gaussian distribution for the sources providing a good match for fMRI spatial components. IVA-GL has been incorporated into the GIFT toolbox (http://mialab.mrn.org/software/gift) and this version of IVA was used in this study.

### Simulation

Previous studies show that inter-subject variability due to different shapes and sizes of the brain that manifest as features such as translation of functional activation sources i.e., variability in location and size of these sources, can be captured through IVA. We hypothesize that this variability can be quantified in the IVA estimated sources of resting fMRI data and attempt to establish the same via simulations. For this, two resting fMRI-like datasets were simulated with three functional activation sources (*C* = 3) representing spatial components in different brain regions with one or two clusters as described in Erhardt et al. (2011, 2012). The data was simulated such that the two datasets had different variance in the translation along the × direction so as to introduce different variability in the spatial maps across the subjects in the given set. Eighty realizations of subject data were simulated in each set by adding subject-specific Gaussian noise. The distinction between the two datasets was that one set had high variance in the translation of sources in x-direction (represented by a normal distribution with 0 mean and a standard deviation of 2) and the other set had a low variance (represented by a normal distribution with 0 mean and a standard deviation of 0.5). The two datasets were treated as two groups for further analyses. The simulated data was then smoothed using a 10 mm Gaussian kernel and then subjected to IVA-GL to estimate four components which were subsequently z-scored and masked as explained in the Supplementary Materials. IVA-GL was modeled with four blind sources so as to allow for noise to be estimated as a separate component in addition to the simulated sources. From the estimated four components, the components encompassing the simulated sources were retained and further analysis was done only on these components.

There are many features of functional activation data that can vary across individuals, and IVA is known to hold onto more of this inter-subject variability than other blind source separation techniques (Ma et al., 2013; Michael et al., 2013). One way that activation differs between individuals is explicitly spatial, specifically in the radial extent of the high-amplitude voxel clusters. There are also less explicitly geometric features such as the raw distribution of voxel amplitudes in a given source component. To quantify such variance, the following measures were calculated for each of the estimated source components from IVA-GL and differences between groups (as simulated based on translational variability in the source) was estimated in these measures.

#### Component level spatial variance (CLSV)

The fluctuation of weights in network voxels of a given subject about the mean will furnish us with higher-order statistical information about the connectivity between voxels within a subject's networks. We calculated the variance of the weights of the three IVA component maps that correspond to the simulated sources for each subject. A two sample *t*-test to test for differences in the group mean of the CLSV was done for each component separately. We believed that this analysis would give us an insight into how amplitude variance relates to translational variance in the sources across subjects.

#### Component volume (CV)

Each component with simulated sources for each subject was separately z-scored and a z-threshold of 2 was applied to individual subject SMs as mentioned earlier. The number of voxels surviving this threshold was counted representing the volume of the component above a *z* = 2 threshold. Difference in the mean component volume between the two simulated groups was calculated using a two sample *t*-test. This test would allow us to estimate if a difference in the extent of the high-amplitude clusters had any implication to the variability across subjects.

### Spatial variability analysis in schizophrenia

Anonymized data was collected from 171 individuals (89 healthy controls age: 38.07 ± 14.03 and 82 schizophrenia patients age: 37.51 ± 11.47), including rs-fMRI acquisition, as part of a center of biomedical research excellence (COBRE http://cobre.mrn.org) project. Informed consent was obtained beforehand according to University of New Mexico Human Research Protections Office protocol. Diagnosis of schizophrenia or schizoaffective disorder (18–65 years) was used as a basis for patient selection using Structured Clinical Interview for DSM-IV axis I disorders. Serial clinical assessments were made and a negative toxicology screen was a prerequisite for scanning schizophrenia patients. Exclusion criteria included a history of mental retardation, neurological disorders including head trauma, or of active substance dependence or abuse within the past year. Healthy controls were recruited from the same geographical location after ruling out Axis I disorders using structured clinical interview for DSM-IV axis I disorders–non-patient edition. One hundred and fifty one volumes of $T_{2}^{*}$ weighted functional images scans were collected on a 3-Tesla Siemens Trio scanner with a 12-channel radio frequency coil for each participant while resting with eyes open. Images were acquired using a gradient-echo EPI sequence with *TR/TE* = 2000/29 (ms) with additional parameters as described in Gopal et al. (2014).

The imaging data was preprocessed using an SPM-based preprocessing pipeline within a neuro- informatics system developed at The Mind Research Network—the collaborative imaging and neuroinformatics suite (COINS) data exchange portal (Scott et al., 2011) [http://coins.mrn.org]. Images were realigned using INRIalign and slice-time correction was applied using the middle slice as the reference frame. Data were then spatially normalized to standard MNI space and resampled to 3 × 3 × 3 mm voxels using the non-linear registration implemented in the SPM toolbox. Finally, data were smoothed using 10 mm FWHM Gaussian kernel. The GIFT toolbox (http://mialab.mrn.org/software/gift/) was used to perform IVA-GL on the preprocessed fMRI data that is of the form [*T* (time) × *V* (voxels)]. A relatively high model order [*C* (define *C*) = 75] was used for analysis. Component selection and masking was done as explained in the Supplementary Materials (Gopal et al., 2016). Further statistical analyses were done on IVA spatial maps which were normalized via z-scoring (*z*-threshold = 2) for each subject for only non-artifactual components. Multiple measures of spatial variability as ascertained to be meaningful in simulations above were computed and differences between schizophrenia patients and healthy controls were estimated based on these measures. Statistical tests were used to quantify these differences and these measures and tests performed are described below. Additional measures were also calculated to further explore the variability in the spatial features of component activation clusters as described below.

#### CLSV

As described above, CLSV (voxel amplitude variance in a component map) for each subject was computed for each of the non-artifactual components identified. A two sample *t*-test to test for differences in the group mean of the CLSV was done for each component separately. This test was expected to help in verifying the hypothesis that the variance across the amplitude weights has a bearing to the variability across subjects manifesting as between group differences. Bonferroni's correction was done to correct for multiple comparisons. The correlation between the absolute frame displacement characterizing subject motion estimated from the realignment step and CLSV were also computed to quantify the relationship between spatial variance at head motion of the patients.

#### MATRICS

The National Institute of Mental Health (NIMH) Initiative, Measurement, and Treatment Research to Improve Cognition in Schizophrenia (MATRICS) scores were used to characterize cognitive abilities of all participants. These tests provided us with seven measures of cognitive performance for each individual that included—speed of processing, attention/vigilance, working memory, verbal learning, visual learning, reasoning, and problem solving, social cognition. Correlation between the MATRICS scores and the CLSV were computed to find relationship between variance and trait of schizophrenia patients' performance. Additionally, to quantify the heterogeneity within the cognitive abilities or performance of schizophrenia patients with respect to healthy controls, difference of variance *F*-tests were computed for each of the seven MATRICS scores (healthy controls—schizophrenia patients).

#### CV

As was done for the simulations, the number of voxels that survived a z-threshold of 2 were counted which accounted for the component volume for each subject. Differences in the group mean (healthy controls—schizophrenia patients) for this volume were calculated using a two sample *t*-test for each component which were corrected for multiple comparison using Bonferroni's method. The correlation between the CLSV and CV was calculated across subjects to explore if the extent of clusters of a particular IVA source is related to the amplitude variance in that source.

#### Distance of component peak from centroid (DPC)

Variance in the location of peak (i.e., maximum weight/amplitude in the network maps across subjects) are expected to further shed light on geometrical differences in IVA estimated sources possibly reflecting a translation in the cluster itself and can reveal group differences in stability of component peak locations. The location of maximum amplitude (weight) was computed within the masked component map for each subject in [x, y, z] co-ordinates for each of the 27 components. The centroid for these three-dimensional locations was obtained for each component following which the distance of each subjects' peak location from the centroid location was calculated. Differences in the group mean in the distance of the peak from the centroid were calculated using two sample *t*-tests for each component. Additionally, to assess whether the schizophrenia patients were more widely spread in the location of the peak around the centroid than the healthy controls, a difference of variance *F*-test was done. The *p*-values were Bonferroni corrected for the number of components (Perlstein et al., 2001).

## Results

### Simulations

Similar to previous results from simulations to test IVA-GL, we were able to estimate the source components effectively in our simulation. Three out of the four components represented the sources simulated and were used for further tests. We observed that the two simulated groups showed significant differences in CLSV favoring the group with lower variance in all the three components (*p* < 0.05). We also observed that differences in component volume that survived a z-threshold of 2 exist in all the three components again favoring the group with lower variance (*p* < 0.05).

### Spatial variability analysis in schizophrenia

Of the 75 components, 27 were found to be non-artifactual components representing networks that have been previously implicated in schizophrenia studies. These were categorized into relevant networks based on visual inspection of the location of clusters and are displayed in the figure in the Supplementary Materials. Measures of spatial variance were computed on these 27 non-artifactual components as capsulated in the methods section above. The results of these tests are described as follows.

#### CLSV

**Figure 1:**
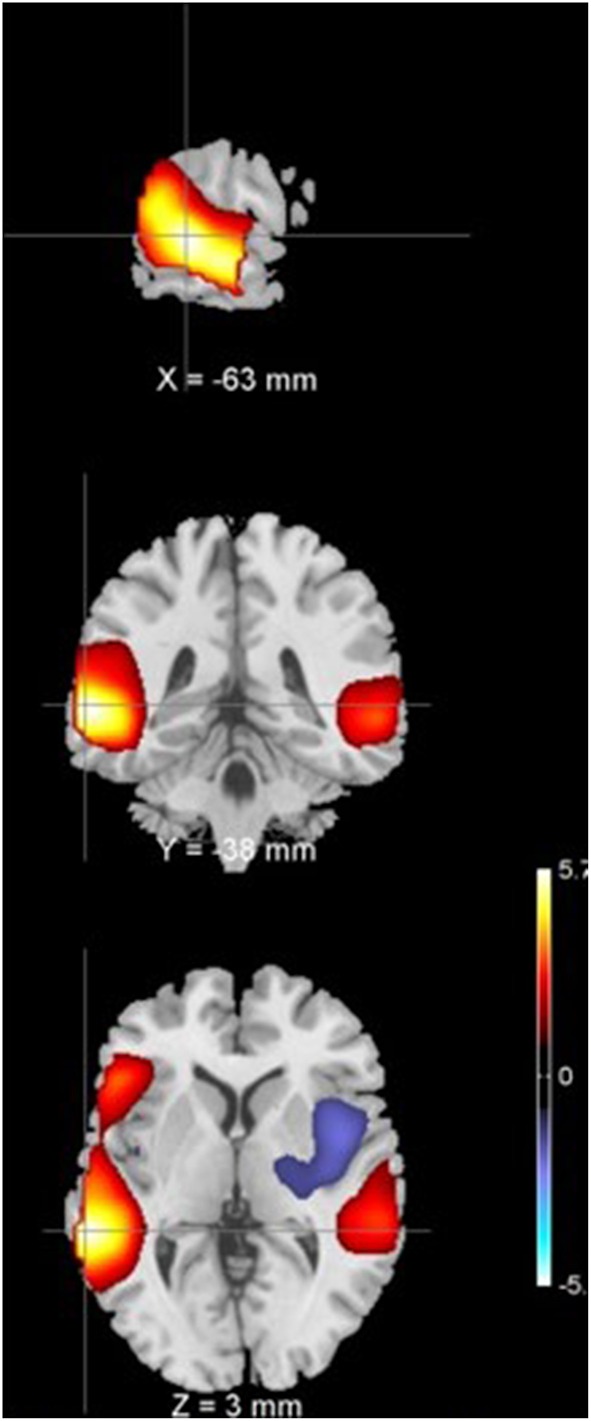
**Component 12 z-scored t-map with a z-threshold of 2 representing middle and superior temporal gyrus**.

**Figure 2:**
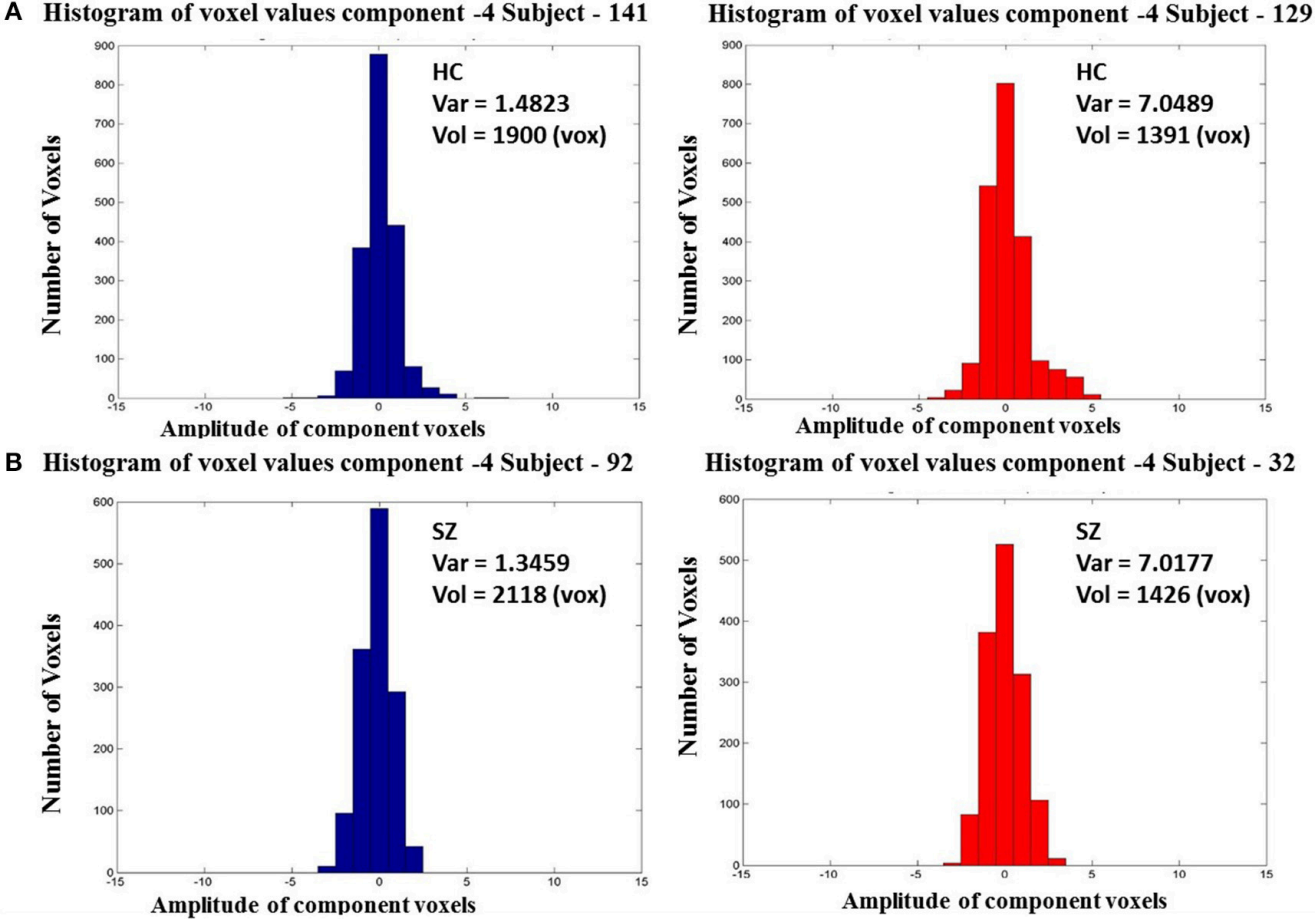
**Histogram of vowel weights or amplitudes of components 4 for two HC's (A) and two SZ's (B) one with high and one with low CLSV**.

It was found that one of the 27 components with *p* = 0.0009 survived multiple comparison correction using Bonferroni's correction at *p* < 0.0019. This component had greater group mean of CLSV for schizophrenia patients than healthy controls and represents areas in the superior and middle temporal gyrus of the auditory network C12 and as shown in Figure 1. These areas are involved in language processing, mathematical operations, recognition of faces, perception of emotion in facial stimuli and word meaning association. Additionally, it was observed through visual inspection that in subjects with lower within subject spatial variance, the histogram of voxel amplitudes (*z* > 2) was flatter i.e., fewer voxels occupied higher amplitudes or weights in the IVA component maps. These components were also observed to have a greater extent of the clusters i.e., a larger number of voxels survived the z-threshold. This was true for both healthy controls and schizophrenia patients. Figure 2 presents the histogram of four subjects—two healthy controls and two schizophrenia patients each with one subject each with high CLSV and one with low CLSV to further elucidate this observation. It was also found that four of the 27 components (C 15—Frontal, C 19—Cingulate, C 20—Visual, and C 23—Frontal) showed statistically significant correlation between the absolute frame displacement and CLSV implying that motion is one of the causes for variance to be introduced into the data. It could be supposed that other sources exist that introduce spatial variance in the data since only some of the components show relationship with frame displacement.

#### MATRICS

It was found, that CLSV of component 7 representing parts of the attention network showed statistically significant negative correlation with the attention/vigilance score. This primarily implies that a greater spatial variance was associated with a lower attention score supporting our hypothesis that schizophrenia patients (with lower attention score) have higher CLSV compared to healthy controls (with higher attention score). No other correlations were found. Moreover, variance differences between healthy controls and schizophrenia patients were found in three of the seven MATRICS measures of cognitive abilities (processing speed, attention/vigilance and reasoning and problem solving) to be significantly different (*p* < 0.05) and the healthy controls had higher scores than schizophrenia patients in all of them. These results are summarized in Table 1.

**Table 1:** **Difference of variance *F*-test results to quantify heterogeneity in cognitive performance of schizophrenia patients and healthy controls**.

**MATRICS category**	**Difference of variance *F*-test**		
	***h***	***p***	**fstat**
Processing speed	1	0.00179	0.49088
Attention vigilance	1	0.00058	0.44759
Working memory	0	0.06736	0.66069
Verbal learning	0	0.34006	0.80607
Visual learning	0	0.17322	0.73482
Resoning and problem solving	1	0.04825	0.63533
Social cognition	0	0.09946	0.68862
Overall composite score	1	5.80E–05	0.38388

#### CV

Three of these eight components representing the sensorimotor network, the visual area components, and the posterior cingulate region of the default mode network, showed significant differences in the mean CV between groups which did not survive multiple comparison correction. They were all unimodal components, i.e., presented one contiguous high-amplitude voxel cluster in each component.

Of the 27 components, 19 were found to have statistically significant correlation between CLSV and CV (*p* < 0.0019 with Bonferroni's correction). Of these 16 components showed a negative correlation, while three components showed positive correlation between CLSV and CV. Table 2 presents the correlation and *p*-values for these 19 components.

**Table 2:** **Correlation r and *p*-values (FDR corrected)**.

**Component**	**Functional area**	***r***	***p***
1	Visual	−0.5054	0.0000
2	Auditory	−0.5139	0.0000
3	Auditory		
4	SMN		
5	SMN	−0.2411	0.0015
6	Visual	−0.7486	0.0000
7	Attentional		
8	DMN–anterior cingulate	−0.5707	0.0000
9	Attentional	−0.2614	0.0006
10	Attentional		
11	Attentional	−0.3386	0.0000
12	Auditory		
13	Basal Ganglia	−0.3341	0.0000
14	DMN−posterior cingulate / precuneus	0.4035	0.0000
15	Frontal		
16	Attentional	−0.3558	0.0000
17	Visual	−0.6685	0.0000
18	SMN	−0.6646	0.0000
19	Cingualte−posterior cingulate/precuneus/cuneus	−0.7288	0.0000
20	Visual	−0.7675	0.0000
21	Attentional		
22	Visual	−0.5370	0.0000
23	Frontal	−0.2386	0.0017
24	Frontal		
25	Cingualte	−0.6687	0.0000
26	DMN—Anterior cingulate	0.6202	0.0000
27	Fontal	0.6013	0.0000

#### DPC

**Figure 3:**
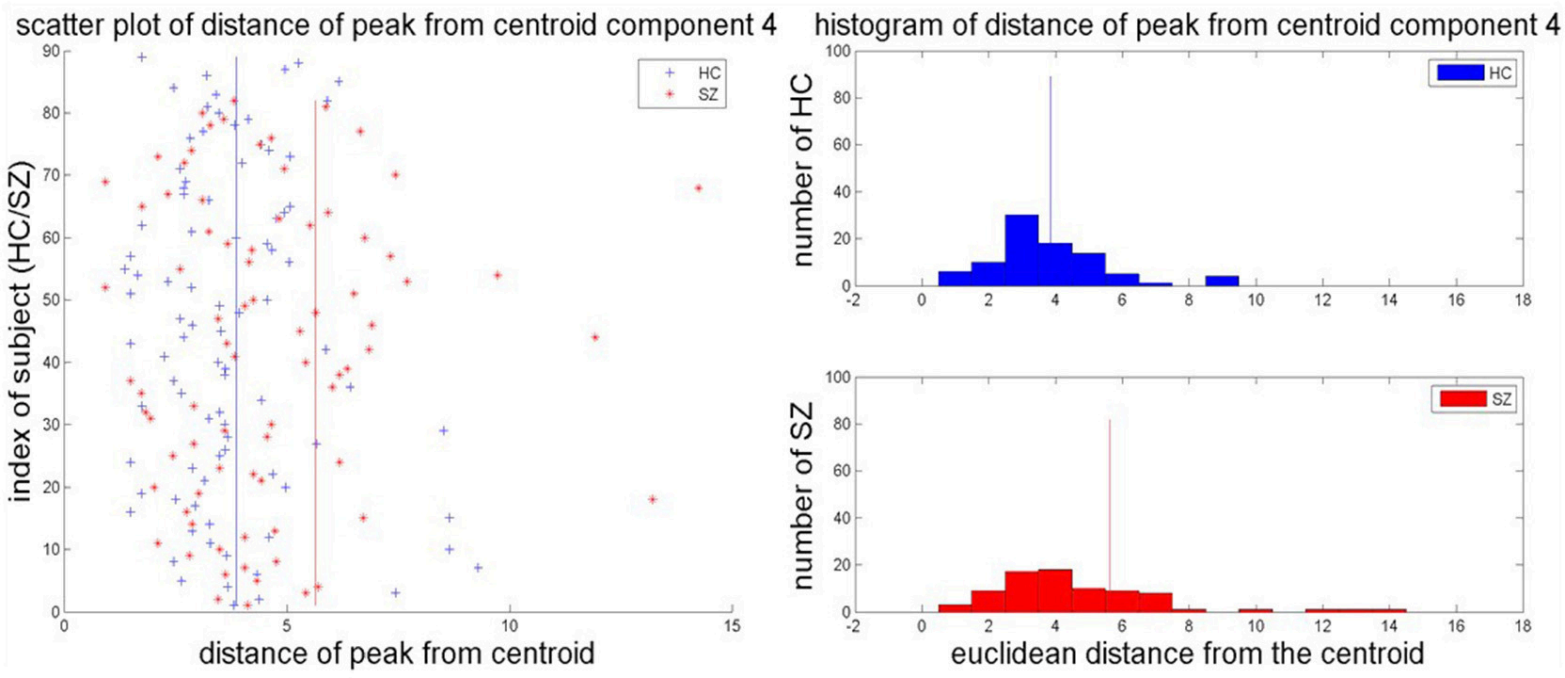
**Scatter plot and group histograms of DPC**. The group mean DPC is represented as red and blue lines for SZ and HC, respectively.

Of the 27 components, two components showed significant differences in the group mean of DPC at *p* < 0.05 but did not survive Bonferroni correction. Thirteen of the 27 components showed differences in the group variance of DPC (*p* = 0.05; schizophrenia patients > healthy controls), of which seven survived Bonferroni correction at *p* < 0.0019. Three of these seven components favored healthy controls (C5—SMN, C11—attentional network, and C25—Cingulate), while the other four favored schizophrenia patients (C1—Visual, C4—SMN, C8—Attention, C21—Attentional, and C23—Frontal). Figure 3 shows the scatter plot of the distance of each subject's peak from the centroid and the histogram of distances of individual subject peaks from the centroid for each group for one representative component C4.

## Discussion

Functional activation patterns during resting state fMRI are known to exhibit individual differences that have an underlying relationship to the cognitive abilities of the individual (Hao et al., 2013; Reineberg et al., 2015). Moreover, each person's brain is unique structurally in the shape, size, and the relative position of sulci and gyri. Analysis of functional imaging data entails averaging functional brain data across subjects. Such analyses assume that pre-processing techniques such as spatial normalization bring homologous areas to approximately a common sub-space. However, there exists a functional localization issue in addition, vis-a-vis variable location of functional sources on the cortical surface of the brain across subjects. Such variability when combined with the anatomical variability may reflect on important functional properties of the brain in terms of cognitive abilities and functional organization of the brain. Characterizing such variability could provide us with valuable insight into what constitutes normal variation, and thereby allow us to explore what constitutes the inherent variability in diseases such as schizophrenia.

Schizophrenia is a cluster of disorders that has been modeled as a disruption in cognitive circuitry manifesting as varied symptoms (Andreasen et al., 1998). This disruption in the cognitive circuitry has been well-studied and is known to be associated with disorganized thinking, disturbed perception, and inappropriate emotions and actions (Freedman, 2007). However, the existence of subtypes in schizophrenia suggests that such cognitive disruption does not present uniformly across the patients. Additionally, previous research into schizophrenia has revealed neuro-anatomical variations within the population such as fluctuations in ventricular and cortical volumes. Such anatomical differences, especially those associated with cortical features and/or localization of functional loci in the brain, might contribute to additional variability in functional activity patterns (Crespo-Facorro et al., 2000, 2009). A structure-function correspondence in spatial variability recognized by studies (Sugiura et al., 2007; Frost and Goebel, 2012; Chechlacz et al., 2015) allows us to perhaps extend variability in functional activation loci to behavior and cognition. When combined with the variability in rs-fMRI activity (which is activity in different regions of the brain relating to activity or thought during rest) we can imagine that resting state networks of functional activation would exhibit a significant amount of variability within the schizophrenia patient population. This study shows that component level variability analyses using IVA, when applied to schizophrenia, elucidate differences between schizophrenia patients and healthy controls that have been previously unexplored. The measures and reported differences in resting-state functional spatial variability within the patient population suggest an important role for higher-order statistical summaries of functional space extending our understanding of this complex disorder.

Schizophrenia patients present lower component-level amplitude variance across subjects in the component representing the middle and superior temporal gyrus, which could be attributed to the fact that they have impaired higher cognitive abilities associated with structural abnormalities in the middle and superior temporal gyrus (Pearlson, 1997; Gaser et al., 2004). However, there are other components representing clusters in the sensorimotor network, the visual network and the anterior and posterior cingulate regions in which healthy controls have greater component level amplitude variance. Even though the differences are not statistically significant, further exploration might result in identifying differences between them that signify different spatial characteristics in healthy controls and schizophrenia patients. This component level amplitude variance is also seen to be related to the component volume. Correlations across subjects indicate a negative relationship between the CLSV and CV for each of eight sub-components. This, in turn, tells us that the extent of individual clusters might play an important role on the variance of the data and thereby the variability of the dataset. Moreover, the inverse relationship between CLSV for attention network component and the MATRICS score for attention/vigilance further bolsters the concept that schizophrenia patients have higher spatial variance associated with lower attention abilities.

The differences in the mean DPC, i.e., in the distribution of the component peaks, further reinforces this result since no component showed statistically significant differences in the mean distance of the subject's peak from the group centroid between healthy controls and schizophrenia patients. However, seven components showed statistically significant difference in the group variance in this measure. This may imply that the spread in the location of the peak about the centroid is characterized by the spatial location of the network in question in conjunction with the effect of disorders such as schizophrenia on those networks. The histogram of the distances for each group has also been plotted in Figure 3. The histogram for schizophrenia patients is right tailed which might indicate that a greater subset of schizophrenia patients tend to have component peaks farther away from the centroid than healthy controls thereby driving up the group mean. This also suggests that the spatial localization of the sensorimotor network is more varied in schizophrenia patients. Simulations to present a similar variability in the extent of clusters replicated results showing that inter-subject variability could be driven by variance in the extent of source components.

Simulations presenting translation in the x-direction show that the variance exists in the periphery of the component. We can thus infer that even slight translation in the clusters would induce significant variance in the data and this could present a reasonable justification to further explore the relationship between spatial variability and other factors such as diagnosis or cognitive abilities, etc. To further bolster this inference, a visual examination of the relationship between CLSV and CV for the components from both simulations as well as real data shows that the variability exists primarily in the extent of the clusters. These observations were also well founded in that evidence of CLSV having statistically significant correlation with absolute frame displacement that characterizes subject motion during the scan was found. It is however interesting to note that these were primarily restricted to visual areas and frontal areas of the brain and possibly imply the presence of other sources of variance in the data in the other spatial areas of the brain. Visual evidence of lateralization was also observed that warrant further inspection to evidence this effect as it might entail additional variability specifically in the DPC measure and reducing the strength of the differences observed.

The results from analyzing differences in component level spatial variability in activation patterns bring to light previously unidentified differences in complex networks affecting schizophrenia patients and healthy controls. The different direction of difference in variance of the DPC i.e., schizophrenia patients > healthy controls or healthy controls > schizophrenia patients, as well as the division of networks with positive or negative correlation between CLSV and CV show that these spatial features of blind source separated spatial components are characterized by the network these components belong to. This relationship is further explained by Figure 2 wherein we can see that subjects with a higher CLSV have a lesser number of voxels occupying higher amplitudes. These results from simulations and functional imaging data provide us with one possible cause for inter-subject variability in functional activation patterns, namely that they result from spatial translation of functional regions on the cortical surface. A detailed look at the components that have positive vs. negative correlations strengthens the motivation to use blind source separation techniques to segregate components that can help illustrate spatial differences between schizophrenia patients and healthy controls.

Based on these results, we can see that spatial variance measures present us with previously unidentified differences between schizophrenia patients and healthy controls. They also present us with ways of identifying differences previously uncharacterized by other analyses techniques in previous studies of schizophrenia. The areas implicated have all been previously associated with imaging as well as non-imaging studies of schizophrenia (Woodruff et al., 1997). The identification of variability in language areas and areas of the brain involved in higher activities is supported by other studies such as those by Mueller et al. (2013) and Gao et al. (2014) as a network that is affected developmentally even in normal individuals. This might imply that areas that mature late developmentally will exhibit greater variability across subjects. Also consistent with results in these studies is the presence of moderate to low variability in default mode network components. These results present a fresh new approach with multiple measures to differentiate schizophrenia patients from healthy controls and further broaden our understanding of this disorder. We have nevertheless, only begun to explore this avenue and believe that there is much more to be learned about clinical conditions by studying higher order statistical features of network spatial maps.

## Author contributions

The research was conducted by SG as part of her Ph.D. research under the guidance of advisors SB and VC. RM was involved in providing additional assistance in analysis and writing the paper.

## Funding

This work was supported by National Institutes of Health's Center of Biomedical Research Excellence grant number P20GM103472, the National Science Foundation's grant number 1539067 and R01EB005846.

### Conflict of interest statement

The authors declare that the research was conducted in the absence of any commercial or financial relationships that could be construed as a potential conflict of interest.
