# Supplementary material for: Approaches to Capture Variance Differences in Rest fMRI Networks in the Spatial Geometric Features: Application to Schizophrenia
Source: Front Neurosci. 2016 Mar 7;10:85. doi: 10.3389/fnins.2016.00085 (PMC4779907; doi:10.3389/fnins.2016.00085)

**Supplementary Material**

**Patient Selection and Demographics:** The schizophrenia patients recruited lie in a relatively narrow breadth of the spectrum encompassing the population classified as schizophrenia. The patients recruited were non-symptomatic for the major part primarily referring to patients with medication based stability. Nicotine, alcohol and drug abuse were reasons for exclusion from the study. The details of the healthy controls and the schizophrenia patients recruited are presented below.

|  | **HC** | **SZ** |
| --- | --- | --- |
| **Age** | 37.51±11.47 | 38.07±14.03 |
| **Gender** | 63 were males and 26 females | 65 were males and 17 females |
| **Ethnicity** | 52 Not Hispanic or Latino and 37 Hispanic or Latino | 47 Not Hispanic or Latino and 35 Hispanic or Latino |
| **Race** | 80 White; 2 American Indian/ Alaska Native and 7 Black or African American | 69 White; 2 American Indian/ Alaska Native; 9 Black or African American and 2 Asian |
|  |  | 10 Schizoaffective and 72 Schizophrenia |

**Clinical information:**

**Summary of MATRICS cognitive scores and differences between HC and SZ.**

|  | **One sample HC** | | | | **One sample SZ** | | | |
| --- | --- | --- | --- | --- | --- | --- | --- | --- |
| **MATRICS Category** | **h** | **p** | **tsat** | **sd** | **h** | **p** | **tsat** | **sd** |
| **Processing Speed** | 1 | 1.93E-67 | 57.4995 | 8.3832 | 1 | 4.45E-39 | 25.5727 | 11.9652 |
| **Attention Vigilance** | 1 | 7.48E-58 | 46.8922 | 9.3006 | 1 | 6.87E-36 | 23.145 | 13.9017 |
| **Working Memory** | 1 | 1.39E-57 | 43.0536 | 10.1983 | 1 | 5.10E-41 | 27.2940 | 12.5467 |
| **Verbal Learning** | 1 | 2.54E-64 | 52.4987 | 7.8040 | 1 | 9.73E-52 | 38.6633 | 8.6922 |
| **Visual Learning** | 1 | 3.75E-54 | 38.8702 | 10.7191 | 1 | 3.10E-39 | 25.7090 | 12.5046 |
| **Resoning and Problem Solving** | 1 | 2.48E-67 | 59.4154 | 8.3529 | 1 | 1.26E-48 | 35.9759 | 10.4795 |
| **Social Cognition** | 1 | 4.97E-59 | 44.93556 | 10.46426 | 1 | 2.31E-42 | 28.53977 | 12.61004 |
| **Overall Composite Score** | 1 | 1.69E-59 | 51.1539 | 8.376866 | 1 | 2.49E-31 | 19.99058 | 13.52016 |

| **Difference of mean t-test** | | | |
| --- | --- | --- | --- |
| **MATRICS Category** | **h** | **p** | **Tstat** |
| **Processing Speed** | 1 | 0.00000000 | 11.2618 |
| **Attention Vigilance** | 1 | 0.00000000 | 6.69829 |
| **Working Memory** | 0 | 0.00000053 | 5.23222 |
| **Verbal Learning** | 0 | 0.00000037 | 5.30761 |
| **Visual Learning** | 0 | 0.00000103 | 5.08557 |
| **Resoning and Problem Solving** | 1 | 0.00000000 | 7.87751 |
| **Social Cognition** | 0 | 0.00000002 | 5.95308 |
| **Overall Composite Score** | 1 | 0.00000 | 9.81610 |

Component selection: Non-artifactual group components were identified using the low/high frequency spectral power measure as a guide^28^ along with visual inspection from the GICA group components. The group averaged z-scored t-test based statistical maps of IVA-GL components and GICA group t-test based maps of components were vectorised. These vectors were correlated to find the IVA components that corresponded to the non-artifactual GICA components identified. The correlation between GICA group components and group averaged IVA components was done with replacement. To elaborate, each GICA group component was correlated with all IVA group components and the pair with maximum correlation was picked. This created the possibility of having multiple GICA group components having maximal correlations with the same IVA group component. For example component 26 of the IVA sources presented as component 21 in figure 1 of supplementary material correlated with component 56 of GICA group component presented as component 21 in figure 1 of supplementary material as well as component 12 of GICA component. The component selection was also dependent on the correlation value i.e. a threshold of 0.6 was used and only component pairs with correlation values higher than that were used. Once these were found a mask was generated for each of the non-artifactual component pairs identified.

Masking: The mask consisted of voxels that passed a z-threshold of 2 in either the z-scored t-test based group GICA component maps or the corresponding group averaged z-scored t-test based IVA component maps. This made sure that the mask had a larger extent than either the group GICA or the group-averaged IVA component maps. The current threshold of z = 2 was selected based on visual inspection wherein the criterion was to obtain clear separate clusters with minimal speckling in the images for a maximum number of components. The selection was made after testing different z thresholds (z = 1.5, 2.5 and 3) with similar source clustering in the components. Furthermore, there are previous studies using multivariate analyses techniques similar to IVA i.e. GICA using a z-threshold of 2 to support our decision. The masks used for 4 of the 27 components are presented in the figure 2 below.

List of component labels

| Component Pair number | Label |
| --- | --- |
| 1 | Visual |
| 2 | Auditory |
| 3 | Auditory |
| 4 | SMN |
| 5 | SMN |
| 6 | Visual |
| 7 | Attentional |
| 8 | DMN |
| 9 | Attentional |
| 10 | Attentional |
| 11 | Attentional |
| 12 | Auditory |
| 13 | Basal Ganglia |
| 14 | DMN |
| 15 | Frontal |
| 16 | Attentional |
| 17 | Visual |
| 18 | SMN |
| 19 | Cingualte |
| 20 | Visual |
| 21 | Attentional |
| 22 | Visual |
| 23 | Frontal |
| 24 | Frontal |
| 25 | Cingualte |
| 26 | DMN |
| 27 | Fontal |


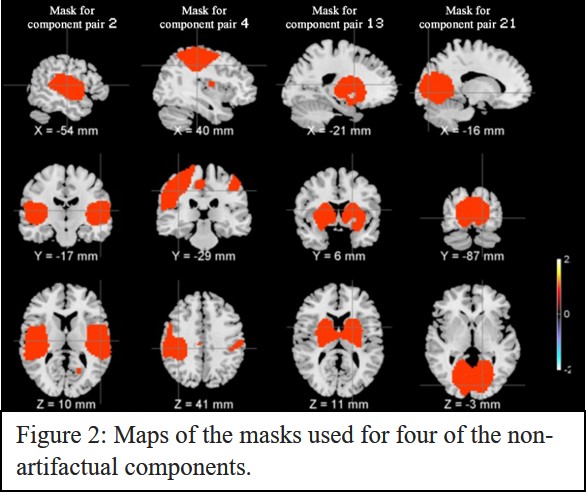


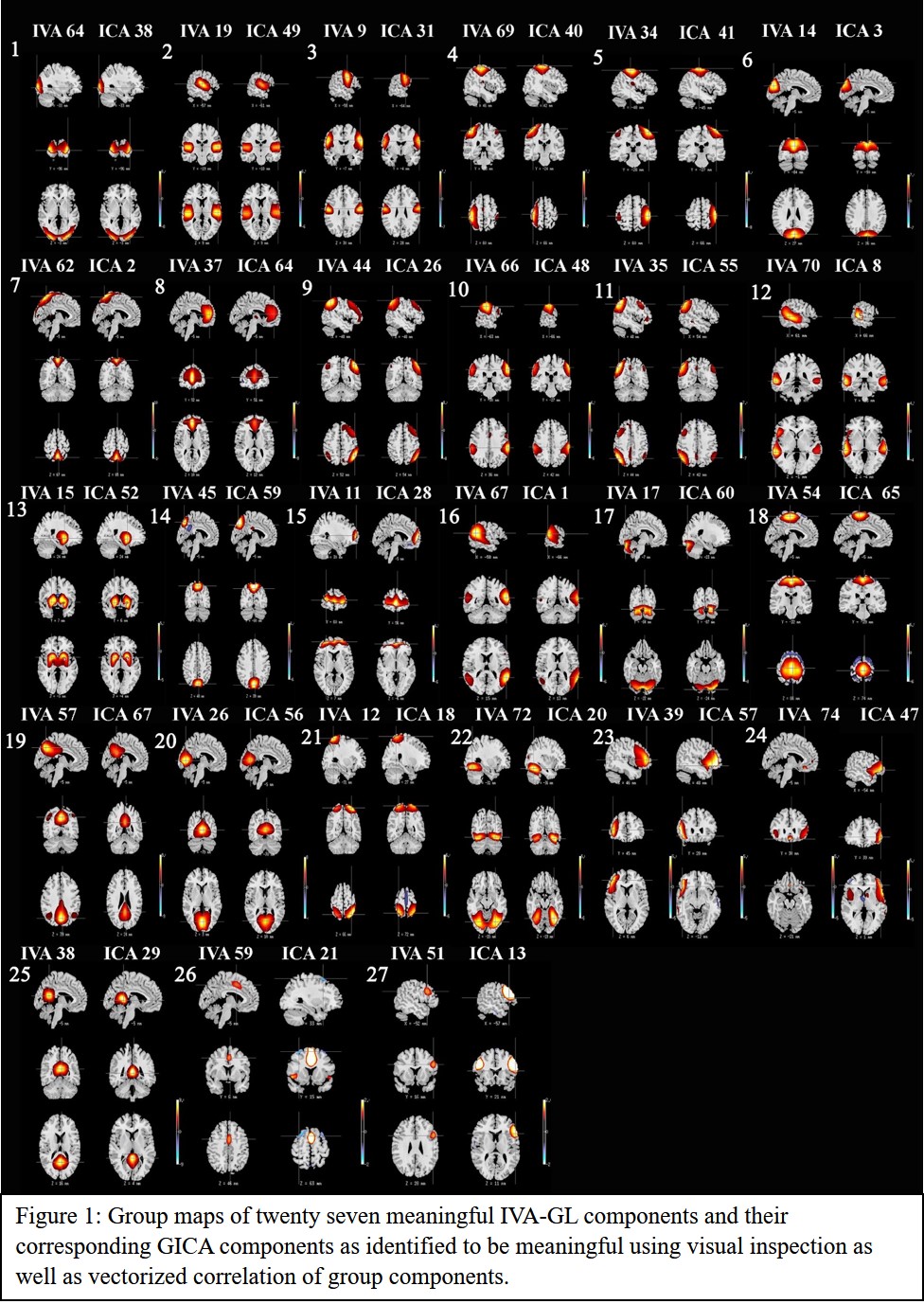

Supplement: Supplementary file 1 [file DataSheet1.DOCX]
